# EGFR blockade confers sensitivity to cabozantinib in hepatocellular carcinoma

**DOI:** 10.1038/s41421-022-00425-y

**Published:** 2022-08-23

**Authors:** Xuhui Ma, Shanshan Wu, Botai Li, Qianqian Zhang, Jianming Zhang, Wenming Liu, Hexin Yan, René Bernards, Wenxin Qin, Cun Wang

**Affiliations:** 1 State Key Laboratory of Oncogenes and Related Genes, Shanghai Cancer Institute, Renji Hospital, Shanghai Jiao Tong University School of Medicine, Shanghai, China; 2 Shanghai Immune Therapy Institute, Renji Hospital, Shanghai Jiao Tong University School of Medicine, Shanghai, China; 3 National Research Center for Translational Medicine (Shanghai), State Key Laboratory of Medical Genomics, Ruijin Hospital, Shanghai Jiao Tong University School of Medicine, Shanghai, China; 4 Department of Anesthesiology, Renji Hospital, Shanghai Jiao Tong University School of Medicine, Shanghai, China; 5 Shanghai Engineering Research Center of Peri-Operative Organ Support and Function Preservation Shanghai, Shanghai, China; 6 Division of Molecular Carcinogenesis, Oncode Institute. The Netherlands Cancer Institute, Amsterdam, The Netherlands

**Keywords:** Cancer genomics, Cancer therapy

Dear Editor,

Current treatment options for patients with advanced hepatocellular carcinoma (HCC) remain limited due to a paucity of drugs that target critical dependencies^1,2^. Broad-spectrum kinase inhibitors such as sorafenib and lenvatinib, which were approved as first-line treatments in clinic, provide a low response rate and modest overall survival benefit to HCC patients^3,4^. For the initial responders, the long-term effectiveness is limited by acquired resistance. It is therefore urgent to develop effective second-line treatments for HCC patients who progressed on first-line treatments.

Since the approval of regorafenib in 2017, the list of second-line treatment candidates has expanded continuously in recent years, which currently contains the monotherapies with regorafenib, cabozantinib, ramucirumab, or pembrolizumab as well as the combination with nivolumab plus ipilimumab. Cabozantinib is an orally bioavailable multi-kinase inhibitor targeting c-MET, c-RET, c-KIT, VEGFR, TIE2 and AXL. The phase III CELESTIAL trial showed that cabozantinib gave a median overall survival/progression-free survival of 10.2/5.2 months compared to 8.0/1.9 months with placebo^5^. Based on the positive data from CELESTIAL trial, cabozantinib has been approved as a second-line treatment for progressed HCC patients who have previously treated with sorafenib^5^. However, these modest survival benefits highlight the substantial clinical need for identifying combination strategies to improve the clinical efficacy of cabozantinib-based therapies. Shang et al. have recently found that cabozantinib treatment led to a strong anti-tumor effect in HCC mouse models caused by Met overexpression^6^. Since about 44% of HCC samples showed co-activation of AKT/mTOR and c-MET in the TCGA LIHC dataset, the combination of pan-mTOR inhibitor MLN0128 and cabozantinib was also determined in c-Met/β-catenin HCC model^6^. However, it is still not clear at present whether this is the most powerful combination for HCC therapy and therefore large-scale genetic screening and compound screening can be helpful for the identification of the most powerful combination therapies with cabozantinib for HCC.

To address this, we treated a panel of HCC cell lines with increasing concentrations of cabozantinib (Detailed methods shown in Supplementary Data S1). Proliferation was impaired in MHCC97H cells, whereas other HCC cell lines displayed no sensitivity to cabozantinib (Fig. 1a). The activities of MET (p-MET) in HCC cell lines before and after treatment of cabozantinib were detected. It is clear that MHCC97H cells have high MET expression which is consistent with previous finding that this cell line has a *MET* gene amplification (Fig. 1b)^7^. Therefore, we sought to identify compounds, which can enhance the effects of cabozantinib in cabozantinib-insensitive HCC cell lines. A kinome-based CRISPR screening was used to systematically identify the kinase whose inhibition confers sensitivity to cabozantinib in Hep3B cells (Fig. 1c). Hep3B cells were infected with the lentiviral kinome guide RNA (gRNA) library and cultured in the absence or presence of cabozantinib for 14 days. Then, genomic DNA was isolated from both untreated and cabozantinib-treated cells. Several independent gRNAs targeting *EGFR* were depleted specifically in cabozantinib-treated group, suggesting that EGFR inhibition is synthetic with cabozantinib (Fig. 1d; Supplementary Table S1). Furthermore, based on a library screen of 2103 compounds in the cabozantinib-insensitive cell line Hep3B (Fig. 1e), a total of three EGFR inhibitors (WZ3146, AST-1306 and poziotinib) were identified, all of which confered sensitivity to cabozantinib in Hep3B cells (Fig. 1f; Supplementary Table S2).

**Fig. 1:**
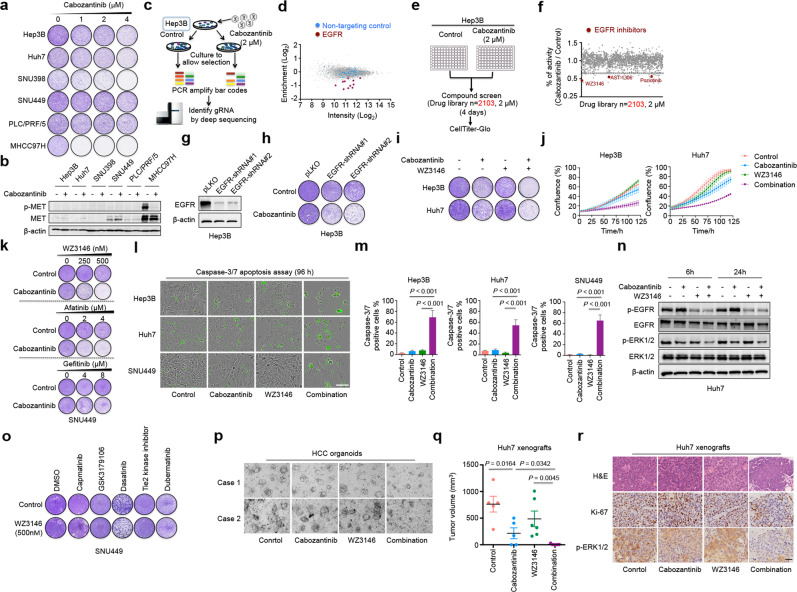
EGFR blockade confers sensitivity to cabozantinib in HCC. **a** Long-term colony formation assays of a panel of liver cancer cell lines treated with increasing concentrations of cabozantinib. **b** Western blot analysis of liver cancer cell lines treated with cabozantinib (4 μM) for 24 h. **c** Schematic representation of the CRISPR-Cas9-based kinome screens performed in Hep3B cells. **d** *EGFR* was identified as a high-confidence synthetic lethal gene with cabozantinib in Hep3B cells. **e** Schematic outline of the compound screen (*n* = 2103). Hep3B cells were screened with compound library in the presence or absence of 2 μM cabozantinib for 4 days. Cell viability was measured using CellTiter-Glo assay. **f** Graph depicting the effects of compounds on cell viability. **g** Hep3B cells were stably transduced with control pLKO vector or with two independent shRNAs targeting *EGFR* (shEGFR#1, shEGFR#2) and the efficiency of knockdown was evaluated by western blot. **h** Colony formation assays of Hep3B cells with and without *EGFR* knockdown were performed in the absence or presence of cabozantinib (4 μM). **i**, **j** Long-term colony formation assays and IncuCyte cell proliferation assays showing synergistic response to cabozantinib (4 μM) combined with WZ3146 (500 nM) in Hep3B and Huh7 cells. **k** Long-term colony formation assays testing the synergistic effect of cabozantinib (4 μM) and FDA-approved EGFR inhibitors (gefitinib and afatinib) on SNU449 cells. **l**, **m** Representative images of Hep3B, Huh7 and SNU449 cells treated with cabozantinib (4 μM), WZ3146 (500 nM) or the combination of both drugs for 96 h in the presence of a green fluorescent caspase-3/7 activatable dye (scale bar = 100 μm). The proportion of cells containing caspase-3/7 staining is shown. **n** Huh7 cells were treated with cabozantinib (4 μM), WZ3146 (500 nM) or the combination of both drugs at the indicated time points prior to western blot analysis with the indicated antibodies. **o** Long-term colony formation assays testing the synergistic effect of WZ3146 (500 nM) and c-MET inhibitor (capmatinib, 4 μM), c-RET inhibitor (GSK3179106, 4 μM), c-KIT inhibitor (dasatinib, 50 nM), TIE2 inhibitor (Tie 2 kinase inhibitor, 4 μM), or AXL inhibitor (dubermatinib, 25 nM), respectively. **p** Representative images of HCC organoids treated with cabozantinib (2 μM), WZ3146 (500 nM) or the combination of both drugs for 8 days. **q** Tumor volumes of Huh7 xenografts in BALB/c nude mice following vehicle, cabozantinib (60 mg/kg), WZ3146 (25 mg/kg) or combination treatment for 14 days. **r** Representative images of H&E, Ki67, and p-ERK stainings performed on formalin-fixed, paraffin-embedded Huh7 xenografts from mice sacrificed after the last dose of vehicle, cabozantinib, WZ3146 or combination treatment (scale bar = 50 μm).

To validate whether EGFR signaling could be responsible for the limited response to cabozantinib, we established *EGFR*-knockdown cell line. shRNA-mediated EGFR knockdown in Hep3B cells sensitized them to cabozantinib, indicating a causal relationship between EGFR signaling and sensitivity to cabozantinib (Fig. 1g, h). Further validation was performed by treating HCC cell lines (Hep3B and Huh7) with a combination of cabozantinib and the EGFR inhibitor WZ3146, which is identified from compound screening. WZ3146 treatment showed a strong synergy with cabozantinib in HCC cells in both long-term and short-term assays (Fig. 1i, j). We also tested the combined effects of cabozantinib and two FDA-approved EGFR inhibitors including gefitinib and afatinib. WZ3146 and afatinib showed obviously stronger synergistic effects with cabozantinib than that of gefitinib on SNU449 cells (Fig. 1k). It is important in this context to point out that these drugs were designed to inhibit mutant EGFR, while the *EGFR* gene in most of HCC cell lines is not mutated. Thus, the affinity of these molecules for wild-type EGFR may differ. Indeed, it appears that WZ3146 and afatinib have stronger effects on EGFR activity in the HCC cell line model as compared to gefitinib (data not shown). This most likely explains the relatively weak effect of gefitinib in combination with cabozantinib.

To address the mechanism by which EGFR blockade and cabozantinib synergize to reduce the viability of HCC cell lines, we measured apoptosis induction in the presence of cabozantinib, WZ3146 or the combination of these two drugs. Monotherapy of these two inhibitors showed modest evidence of apoptosis induction in three HCC cell lines. However, strong synergistic induction of apoptosis was observed when cells were treated with the combination of cabozantinib and WZ3146 (Fig. 1l, m). As mentioned above, WZ3146 was developed as a mutant-selective EGFR inhibitor against EGFR T790M^8^, whereas in our study we observed that WZ3146 also obviously inhibited the wild-type EGFR (Fig. 1n). Moreover, western blot analyses indicated that the combination of cabozantinib and WZ3146 resulted in an increased inhibition of p-ERK in Huh7 cells (Fig. 1n). Then we further explored the potential mechanism of the synergy between WZ3146 and cabozantinib. As we mentioned above, cabozantinib is a multi-kinase inhibitor targeting c-MET, c-RET, c-KIT, VEGFR, TIE2 and AXL. We tested the synergy between WZ3146 and c-MET inhibitor (capmatinib), c-RET inhibitor (GSK3179106), c-KIT inhibitor (dasatinib), TIE2 inhibitor (Tie 2 kinase inhibitor) or AXL inhibitor (dubermatinib), respectively. Only c-KIT inhibitor (dasatinib) shows some synergy with WZ3146 (Fig. 1o). These data suggest that more than one target have been inhibited by cabozantinib to cause synergy between WZ3146 and cabozantinib.

To test whether our in vitro findings could be recapitulated in pre-clinical models, we established two patient-derived HCC organoids for further analyses. The results indicated that cabozantinib or WZ3146 alone slightly suppressed growth of patient-derived organoids, while their combination significantly inhibited cell viability in both HCC organoids, suggesting a strong synergy between cabozantinib and WZ3146 (Fig. 1p). Then, we generated Huh7 xenografts for in vivo analyses. Compared to monotherapy, treatment with the combination of cabozantinib and WZ3146 showed a more effective inhibition of tumor growth (Fig. 1q). The combination treatment elicited an obvious inhibition of p-ERK accompanied with decreased number of Ki67-positive cells (Fig. 1r). Importantly, no obvious side effects were observed in mice treated with the combination of cabozantinib and WZ3146, which indicates a good tolerability of this drug combination.

Cabozantinib combination therapies can potentially be powerful in the treatment of progressed HCC patients. Shang et al. described the combination therapy of pan-mTOR inhibitor MLN0128 and cabozantinib for oncogene-driven HCC murine models^6^. Based on unbiased approaches, we identified the combination of EGFR blockade and cabozantinib as a potential strategy for the treatment of HCC. In a recent study, EGFR inhibition was also found to enhance the efficacy of lenvatinib in liver cancer^9^. However, EGFR inhibition did not enhance the efficacy of sorafenib in clinical studies^10^, indicating that the synergy of EGFR inhibition is selective for only a subset of multi-kinase inhibitors. Since the two drugs identified here have already been used in the clinic or are in clinical development, our findings could be readily tested in clinic.

## Supplementary information

Supplementary Data S1

Supplementary Table 1

Supplementary Table 2

## Data Availability

The original data that support the findings are available from the corresponding author (C.W.) on reasonable request.
