# Supplementary material for: EGFR blockade confers sensitivity to cabozantinib in hepatocellular carcinoma
Source: Cell Discov. 2022 Aug 23;8:82. doi: 10.1038/s41421-022-00425-y (PMC9399229; doi:10.1038/s41421-022-00425-y)
Supplement: Supplementary file 1 — Supplementary Data S1 [file 41421_2022_425_MOESM1_ESM.pdf]

Materials and methods

Human cell lines

The human HCC cell lines, Hep3B, Huh7, PLC/PRF/5, SNU398 and SNU449 were provided by Erasmus University (Rotterdam, Netherlands). MHCC97H was provided by the Liver Cancer Institute of Zhongshan Hospital (Shanghai, China). HCC cells were cultured in Dulbecco's Modified Eagle Medium (DMEM) with 10% fetal bovine serum, glutamine and penicillin/streptomycin (Gibco) at 37°C / 5% CO_2. Mycoplasma contamination was excluded via a PCR-based method. The identities of all the cell lines were confirmed by short tandem repeat (STR) profiling.

Compounds and antibodies

Cabozantinib (S4001) and WZ3146 (S1170) were purchased from Selleck Chemicals. Antibody against Ki67 (ab16667) was purchased from Abcam. Antibodies against p-ERK (no. 4370), ERK (no. 4695), p-EGFR (no. 3777), EGFR (no. 4267), p-MET (no. 3077), MET (no. 8189) were purchased from Cell Signaling Technology. Antibody against β-actin (HRP-66009) was purchased from Proteintech.

Pooled CRISPR screen and data processing

For the design of the kinome CRISPR library, 5971 gRNAs targeting 504 human kinases, 10 essential genes and 50 non-targeting gRNAs were selected. Oligos with gRNA sequences flanked by adapters were ordered from CustomArray (Bothell, Washington, USA) and cloned as a pool by GIBSON assembly in LentiCRISPRv2.1. The kinome CRISPR library was introduced into Hep3B cells by lentiviral transduction. Cells stably expressing gRNA were cultured in the absence or presence of 2 µM cabozantinib for 14 days. The abundance of each gRNA in the pooled samples was determined by Illumina deep sequencing. Single-end reads were trimmed and quality-filtered and then matched against sgRNA sequences from the kinome CRISPR library. Subsequently, read counts of sgRNAs were normalized against total read counts across all samples. For each sgRNA, the fold change value for enrichment was calculated between the cabozantinib treated group and untreated group.

Compounds screen

Cells were screened for sensitivity against a compound library including a panel of 2103 small-molecule inhibitors. Briefly, Hep3B cells were plated in 384-well plates in the absence or presence of 2 µM cabozantinib. All compounds from library were tested in two replicates. Cell viability in each well was determined using the CellTiter-Glo. Then the relative survival of Hep3B cells in the absence or presence of 2 µM

cabozantinib was calculated.

Plasmids

EGFR shRNA vectors were retrieved from the arrayed TRC human genome-wide shRNA collection.

shEGFR#1

TRCN0000121067_CCGGGCTGCTCTGAAATCTCCTTTACTCGAGTAAAGGAGATTTCAGAGCAGCTTTTTG;

shEGFR#2

TRCN0000121068_CCGGGCCACAAAGCAGTGAATTTATCTCGAGATAAATTCACTGCTTTGTGGCTTTTTG;

Long-term cell proliferation assays

Cells were cultured and seeded into 6-well plates at a density of 1-2 × 10^4 cells per well, depending on growth rate, and were cultured in medium containing the indicated drugs for 10-14 days (medium was changed twice a week). Cells were fixed with 4% formaldehyde in PBS and stained with 0.1% crystal violet diluted in water.

Incucyte cell proliferation assay and apoptosis assay

Cells were cultured and seeded into 96-well plates at a density of 1000–1500 cells per well. Twenty-four hours later, drugs were added at indicated concentrations. Cells were imaged every 4 hours in IncuCyte ZOOM (Essen Bioscience). Phase-contrast images were collected and analyzed to detect cell proliferation based on cell confluence. For cell apoptosis, caspase-3/7 green apoptosis assay reagent was also added to culture medium and cell apoptosis was analyzed based on green fluorescent staining of apoptotic cells. For each condition, at least three replicates (50 cells / field) were analyzed.

Protein lysate preparation and western blots

Cells were washed with phosphate-buffered saline (PBS) and lysed with RIPA buffer supplemented with Complete Protease Inhibitor (Roche) and Phosphatase Inhibitor Cocktails II and III (Sigma). All lysates were freshly prepared and processed with BioRad Gel Electrophoresis Systems.

Organoid culture

Tumor cell isolation: Fresh patient-derived specimens were minced, washed with PBS, and incubated with the digestion solution on an orbital shaker at 37 °C. The digestion solution consists of Dulbecco's modified Eagle's medium (DMEM, BasalMedia) containing collagenase D (Roche), DNase I (Sigma), Y27632 (Selleck) and Primocin (InvivoGen). The digestion time depends on the degree of fibrosis and the size of the tissue block. Generally, the tissues were digested for 45-120 min, until most of the cell mass has been suspended. After tissue digestion, DMEM media containing 10% fetal bovine serum was added to the suspension to inactivate collagenase D and cell suspension was then filtered through a 70 μm Nylon cell strainer and spun for 5 min at 300-400g. Cell pellets were washed twice with pre-chilled Advanced DMEM/F12 (Thermo Fisher Scientific) and kept cold. 10 μL of this cell suspension could be counted by Trypan Blue to determine the concentration of live cells.

Hepatoma tissue organoids culture: The pellets were resuspended with optimized hepatoma tissue organoid medium which is composed of Advanced DMEM/F12 supplemented with penicillin/streptomycin (BasalMedia), GlutaMAX-1 (BasalMedia), HEPES (BasalMedia), Primocin, B27 supplement (without vitamin A, BasalMedia), Nicotinamide (Sigma-Aldrich), N-acetyl-l-cysteine (Sigma-Aldrich), recombinant human EGF (Novoprotein), Recombinant human FGF10 (Novoprotein), Recombinant human HGF (PeproTech), Recombinant human Rspondin (Novoprotein), Y27632 (Selleck), A8301 (TargetMol), Oncostatin-M (Sigma-Aldrich), CHIR-99021 (TargetMol). Isolated cells (2×10^5) were resuspended using 100 μL organoid medium and mixed with 100 μL of Matrigel (Corning), Then, cells were seeded on 6-well plates (GreinerBio-one), and fixed in a 37°C incubator for 30 min. After the gel was solidified, 3 mL organoid medium was added to each well, and the culture plates were transferred to the incubator for further culture.

Efficacy evaluation of indicated inhibitors on HCC organoids: Every fifty thousand pellets in logarithmic growth phase were resuspended in organoid media and then seeded on a 24-well plate. Cabozantinib (2 μM), WZ3146 (500nM) or the combination were added in the medium and the inhibitory efficacy of the indicated inhibitors on the formation of hepatoma tissue organoids was observed under the microscope.

Xenografts

All animals were manipulated according to protocols approved by the Shanghai Medical Experimental Animal Care Commission and Shanghai Cancer Institute. Huh7 cells (1×10^7 cells per mouse) were injected subcutaneously into the right posterior

flanks of 6-week-old BALB/c nude mice (male, 5-7 mice per group). Tumor volume based on caliper measurements was calculated by the modified ellipsoidal formula: tumor volume = 1/2 length × width^2. After tumor establishment, mice were randomly assigned to 6 days / week treatment with vehicle, cabozantinib (60 mg/kg, oral gavage), WZ3146 (25 mg/kg, oral gavage) or a drug combination in which each compound was administered at the same dose and schedule as single agent.

Immunohistochemical staining

Formalin-fixed paraffin-embedded samples were obtained from xenograft tumors and then probed with antibodies against Ki67 (ab16667, Abcam) and p-ERK (no. 4370, Cell Signaling Technology). Following incubation with the primary antibodies, positive cells were visualized using DAB+ as a chromogen.
